# Development of a Traditional Chinese Medicine Syndrome-Specific Scale for Ulcerative Colitis: The Large Intestine Dampness-Heat Syndrome Questionnaire

**DOI:** 10.1155/2018/4039019

**Published:** 2018-07-12

**Authors:** Xin-lin Chen, Yi Wen, Zu-chun Wu, Bei-ping Zhang, Zheng-kun Hou, Jun-lin Xiao, Man-qing Lin, Yue Hu, Zhe-li Wu, Jie-min Deng, Feng-bin Liu, Tian-wen Liu

**Affiliations:** ^1^School of Basic Medical Science, Guangzhou University of Chinese Medicine, Guangzhou, China; ^2^The First Clinical College, Guangzhou University of Chinese Medicine, Guangzhou, China; ^3^The Second Affiliated Hospital, Guangzhou University of Chinese Medicine, Guangzhou, China; ^4^The First Affiliated Hospital, Guangzhou University of Chinese Medicine, Guangzhou, China; ^5^The Second Clinical College, Guangzhou University of Chinese Medicine, Guangzhou, China

## Abstract

The aim of this study was to develop and validate the large intestine dampness-heat syndrome questionnaire (LIDHSQ) for patients with ulcerative colitis (UC). The domains and items of the LIDHSQ were developed according to standard procedures, namely, construct definition, item generation, language testing, content validity, pilot study, and validation study. At first, a total of 20 items in 3 domains were generated based on literature review and expert consultation. After the item selection, the LIDHSQ contains 11 items in three domains: disease-related domain (diarrhoea, abdominal pain, bloody purulent stool, and mucus stool), heat domain (fever, dry mouth, red tongue, yellow fur, and anal burning), and dampness domain (greasy fur and defecation disorder). The Cronbach's alphas of all domains were greater than 0.6. All of the intraclass correlation coefficients were greater than 0.8. The LIDHSQ and domain scores of the patients with LIDHS were higher than those of the patients with other syndromes (*P* < 0.001). The area under the receiver operating characteristic curve of the LIDHSQ was 0.900, with a 95% confidence interval of 0.872–0.928. When the cut-off value of the LIDHSQ was ≥ 7, the sensitivity and specificity were 0.867 and 0.854, respectively. The LIDHSQ is valid and reliable for measuring LIDHS in UC patients with good diagnostic efficacy. We recommend the use of the LIDHSQ in Chinese UC patients.

## 1. Introduction

Ulcerative colitis (UC) is an idiopathic, chronic inflammatory disorder of the colonic mucosa that starts in the rectum and generally extends proximally in a continuous manner through either part of or the entire colon. In the West, the incidence and prevalence of UC have increased over the past 50 years, up to 8-14/100,000 and 120-200/100,000 persons, respectively [1]. Some studies have reported that the incidence and prevalence rates of UC have increased gradually in China [2–5]. The incidence of UC varied from 0.07 to 4.90 per 100,000 people per year in China based on the results of a multinational, population-based study conducted between 2011 and 2013 [3]. It was estimated that the prevalence of UC in China is 11.6 per 100,000 people in 2016 [4]. Large intestine dampness-heat syndrome (LIDHS) is one of the most common UC syndromes [6–9]. Chen et al. reported that LIDHS accounted for 34.8% of the UC patients according to the results of a literature review [6].

Traditional Chinese medicine (TCM) is widely used to treat UC as a complementary and alternative medicine [10–16]. Some clinical research has reported that TCM therapies are effective for treating UC patients with LIDHS in the clinic [8, 13, 14]. Chinese herbal medicine can effectively inhibit intestinal inflammation for UC patients with LIDHS [8, 14].

The mainstay of LIDHS diagnosis was based on the experience of TCM doctors. However, the diagnosis of LIDHS is problematic. First, the diagnostic criteria for LIDHS in UC patients are not uniform. Five diagnostic criteria for LIDHS can be found in the literature [17–21]; however, these criteria all contain different items. For example, mucus stool was the main item in three criteria [17, 18, 21] but was not found in the others [19, 20]. The lack of consistent criteria increases the difficulty of diagnosing LIDHS. Second, there is a lack of validated and standardized LIDHS-specific scales in TCM clinical practice.

Some researchers have reported that the standard development and validation procedures for health-related quality of life (HRQOL) or patient-reported outcomes (PRO) questionnaires can be effectively used to develop and assess TCM outcomes and syndromes [22–29]. For example, our team developed and validated TCM outcomes using these procedures [22, 25]. Diagnostic tests are a type of medical procedure performed for the diagnosis of a disease [30, 31] and are also tools used to diagnose TCM syndromes [32]. The aim of this study was to use standard development and validation procedures to develop and assess the large intestine dampness-heat syndrome questionnaire (LIDHSQ) which was administered by the doctors.

## 2. Methods

**Figure 1:**
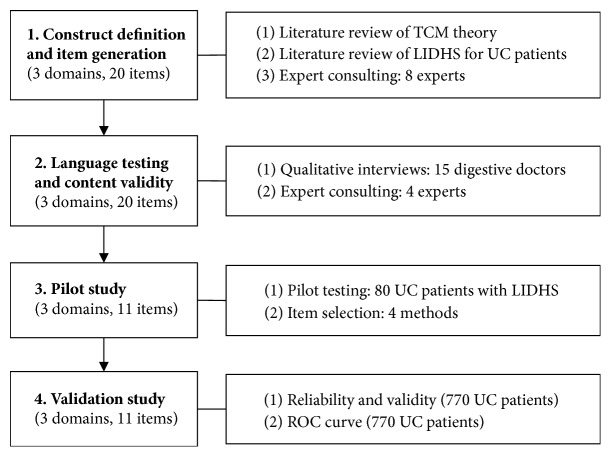
Four steps towards development of the procedure.

The standard procedures for questionnaires were followed to develop and validate the LIDHSQ for UC [33–35]. According to the suggestions of the World Health Organization Quality of Life (WHOQOL) instruments group and the US Food and Drug Administration (FDA), the procedures include the following five steps: (1) hypothesizing the conceptual framework; (2) adjusting the conceptual framework and drafting the instrument; (3) confirming the conceptual framework and assessing other measurement properties; (4) collecting, analysing, and interpreting the data; and (5) modifying the instrument [28, 33–35]. For the development of the LIDHSQ, the procedure included construct definition and item generation, language testing and content validity, a pilot study, and a validation study (Figure 1).

### 2.1. Construct Definition and Item Generation

The LIDHS refers to the syndrome resulting from dampness and heat attacking large intestine, mainly leading to the dysfunction of the large intestine in transportation of food and waste [36]. Dampness is a pathological substance formed by the invasion of external moist pathogen or abnormal movement of body fluid. Heat can be caused by exogenous Yang-heat pathogenic factors, emotional hyperpolarization, or excesses of viscera-Qi. The interaction between dampness and heat can lead to a series of clinical symptoms for UC patients, especially those in the gastrointestinal tract, such as abdominal pain, diarrhoea, bloody stool, mucus stool, and nausea and vomiting. The UC patients with LIDHS usually have some symptoms of dampness (such as greasy fur, slippery pulse, soft pulse, and mucus stool) and heat (such as fever, dry mouth, halitosis, red tongue, and anal burning).

The research team was composed of three TCM physicians, two TCM diagnostics educators, and two HRQOL researchers. A literature review and expert consultation were used to establish the domains of the LIDHS. (1) A literature review on LIDHS for UC was conducted. Five criteria for LIDHS in UC patients in guidelines or guidebooks were found [17–21]. (2) Expert consultation was performed. Five TCM digestive doctors and three experts in TCM diagnosis with over 10 years of clinical research and teaching experience were included. The research team reviewed the related TCM scales such as the Chinese Quality of Life Instrument (ChQoL) and the suboptimal health status questionnaire (SHS). Based on the TCM theory and the results of literature review and expert consultation, the team proposed three domains of LIDHS for UC patients: the disease-related domain, heat domain, and dampness domain. The disease-related domain was used to indicate the location of the disease, which can be used to distinguish it from other dampness-heat syndromes, such as spleen-stomach dampness-heat syndrome. The heat domain was related to the diagnosis of heat syndrome, and the dampness domain was related to the diagnosis of dampness syndrome.

Based on the literature review on LIDHS for UC patients, eighteen items were included (Table 1). These items contained diarrhoea, abdominal pain, bloody purulent stool, mucus stool, tenesmus, anal burning, fever, scanty deep-yellow urine, bitter taste in mouth, dry mouth, halitosis, red tongue, greasy fur, yellow fur, thick fur, rapid pulse, slippery pulse, and soft pulse. The experts in expert consultation suggested that another two items (anorexia and defecation disorder) were common TCM symptoms for the UC patients with LIDHS and might be helpful to improve the sensitivity and specificity of the LIDHSQ. Thus, the two items were added. Finally, a total of 20 items were generated and classified into three domains: disease-related domain (five items), heat domain (nine items), and dampness domain (six items). The response options for the items were binary: yes (positive) or no (negative).

### 2.2. Language Testing and Content Validity

All items were tested by 15 TCM digestive doctors. We ensured that the items could be understood by TCM doctors and that the meaning of the item did not cause ambiguity. The content validity of the items was also assessed by these digestive doctors. Problematic items were modified according to their suggestions. Expert consultation (4 experts) was available to evaluate whether the items could represent the most important aspects of the LIDHSQ for UC [37]. Minor revisions and rewording of some items were performed until content validity was achieved. Finally, the first version of the LIDHSQ was established.

### 2.3. Pilot Study

A pilot study (cross-sectional study) was conducted to select the items. The UC patients with LIDHS were enrolled. The Research Ethics Committee of Guangzhou University of Chinese Medicine provided ethical approval. All of the patients provided informed consent to participate. Eligibility criteria included the following: (1) diagnosed with UC according to the criteria drafted by the inflammatory bowel disease (IBD) group of the Chinese Medical Association Gastroenterology Branch (CMAGB) [38], (2) diagnosed with LIDHS according to the criteria drafted by spleen and stomach disease branch of the China Association of Chinese Medicine (CACM) [20], (3) aged ≥ 12 years, and (4) providing informed consent to participate. The patients were excluded if they were diagnosed with other syndromes or other diseases.

The items were screened and selected using the following four methods: (1) The proportion of positive responses for each item was calculated, and the items with proportion < 0.4 were marked. (2) At first, we calculated the internal consistency coefficient (*α* value) of all the items. At each time, one of the items was deleted. And then we calculated the new *α* value and observed the change of *α* value for each item deleted. The item with the change of *α* value > 0 was marked. (3) Correlation coefficient (r) between each item and its corresponding domain was calculated, and the item with r < 0.4 was marked. (4) Confirmatory factor analysis (CFA) of each item was performed, and the items with loading factor < 0.5 were marked. When the item meets two or more of the above criteria, it will be finally removed.

### 2.4. Validation Study

A validation study was conducted to assess the psychological characteristics of the LIDHSQ. The Research Ethics Committee of Guangzhou University of Chinese Medicine provided ethical approval. The patients were enrolled from the First Affiliated Hospital and the Second Affiliated Hospital of Guangzhou University of Chinese Medicine. The inclusion criteria of the patients were the following: (1) diagnosed with UC according to the criterion of the IBD group of the CMAGB [38] and diagnosed with LIDHS or other syndromes [20]; (2) aged > 12 years; (3) not affected by other infectious diseases; (4) and providing informed consent to participate. The exclusion criteria were the following: (1) diagnosed with other diseases (e.g., Crohn's disease); (2) having another severe disease or complicated polymorbidities with polar outcomes, such as cancer, cardiovascular disease, diabetes, renal disease, or liver disease; and (3) being pregnant or nursing.

The experienced TCM doctors (working for more than 5 years) evaluated the syndromes (LIDHS and other syndromes) of UC patients according to the criteria drafted by the spleen and stomach disease branch of the CACM [20]. To ensure the consistency and accuracy of the TCM syndromes for UC patients, the syndrome of each patient was diagnosed by two experienced doctors. If there was inconsistency, a third chief doctor (associate professor or above) resolved the disagreement. The diagnostic results of the doctors were considered as the gold standard.

All of the patients were also assessed by the TCM doctor using the LIDHSQ. Twenty-two inpatients were assessed using the LIDHSQ within 7–10 days, which was used to assess test-retest reliability. The patients completed a sociodemographic form without assistance. The sociodemographic form covered age, sex, marital status, occupation, smoking, drinking, dietary preferences, and family history.

SPSS for Windows (version 22.0) and Lisrel software (version 8.7) were used for the analysis. The Kappa coefficient was applied to calculate the agreement between the two experts. The percentage of missing data and the frequency of each item were described. The correlation coefficients between the item and its own domain or other domains were calculated. Cronbach's *α* value was used to evaluate internal consistency reliability. Test-retest reliability was assessed using the intraclass correlation coefficient (ICC) and its 95% confidence interval (CI). Construct validity was assessed using root mean square error of approximation (RMSEA) and some fit indexes based on structural equation modelling (SEM) [39, 40]. The domain scores between patients with LIDHS and those with other syndromes were compared using* t*-tests. Diagnostic tests were used to test the diagnostic value (sensitivity and specificity). The cut-off value was also calculated.

## 3. Results

### 3.1. Pilot Study

Eighty UC patients with LIDHS were included in the pilot study (Table 2). The mean age of the patients was 48.3 (range: 18–85) years. Fifty patients were male, and 75 were married.

The results of item selection were shown in Table 3. After item selection, nine items, namely, tenesmus, scanty deep-yellow urine, bitter taste in mouth, halitosis, yellow fur, thick fur, rapid pulse, slippery pulse, and soft pulse, were deleted. The LIDHSQ for UC patients contained 11 items covering three domains: The disease-related domain included diarrhoea (item 1), abdominal pain (item 2), mucus in stool (item 3), and bloody purulent stool (item 4). The heat domain included anal burning (item 5), red tongue (item 6), yellow fur (item 7), fever (item 8), and dry mouth (item 9). The dampness domain included greasy fur (item 10) and defecation disorder (item 11). The scores on each domain (or the LIDHSQ) were summed to yield a score for the domain (or the LIDHSQ). The final version of the LIDHSQ is shown in the Supplementary Materials (Table S1).

### 3.2. Validation Study

A total of 780 UC patients were included in the validation study. Ten patients refused to participate in the study due to limited time. In all, 770 UC patients were included (391 males and 379 females) (Table 2). The mean age of all of the patients was 46.0 (range: 12–86) years. There were 249 patients with LIDHS and 521 patients with other syndromes. The proportion of sexes between patients with LIDHS and those with other syndromes was significantly different (*P* < 0.001). There were no significant differences in age, marital status, occupation, smoking, drinking, dietary preferences, or family history between the two groups (*P* > 0.05).

None of the items had missing data (Table 4). The item “abdominal pain” scored the highest (0.936), followed by diarrhoea (0.928) and red tongue (0.900). The item “fever” scored the lowest (0.402). All of the Kappa coefficients between the two experts were greater than 0.9 (Table 4). The correlation coefficients of each item with its own domain (*P* < 0.001) were greater than those of each item with other domains.

The Cronbach's *α* value of the LIDHSQ was 0.68 (Table 5). The *α* values of all domains were greater than 0.6 and were statistically significant (*P* < 0.05). All of the ICCs were greater than 0.8 and were significantly different (*P* < 0.05, Table 5). All domain scores and LIDPSQ scores of the patients with LIDPS were higher than those of the patients with other syndromes. All of the scores between the two groups were significantly different (*P* < 0.001, Table 6).

**Figure 2:**
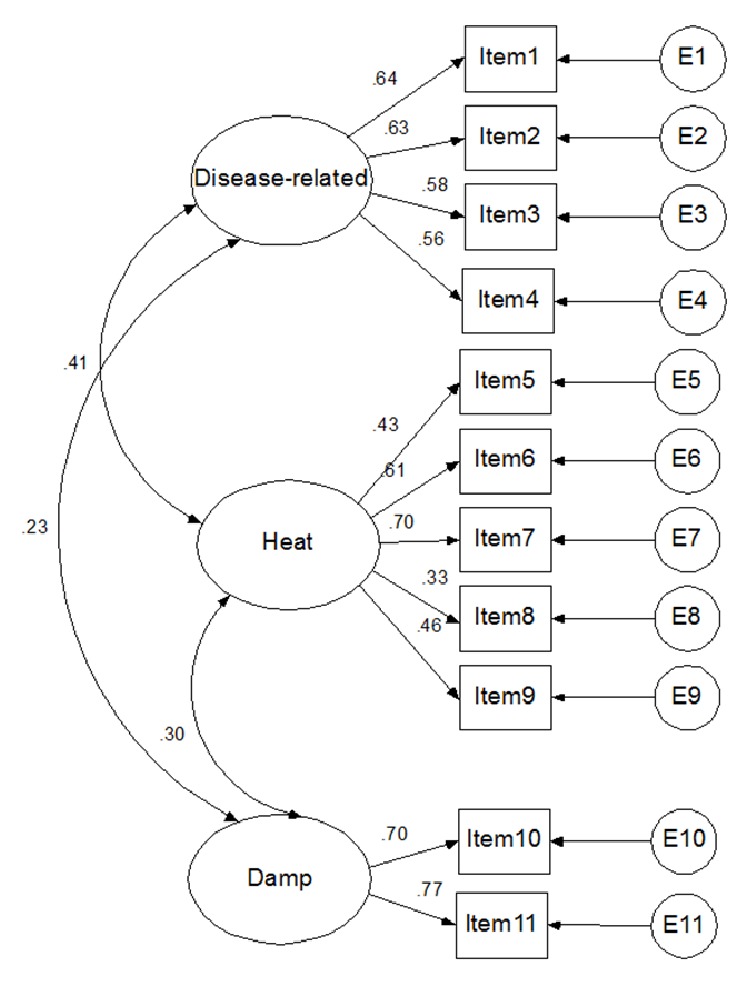
Structure diagram (standardized coefficient) of large intestine dampness-heat syndrome (items 1-11: diarrhoea, abdominal pain, mucus stool, bloody purulent stool, anal burning, red tongue, yellow fur, fever, dry mouth, greasy fur, and defecation disorder).

The chi-square value was 123.8 (*P* = 0.001) based on the SEM results. The RMSEA was equal to 0.090 (90% CI: 0.072–0.109). The comparative fit index (CFI), normed fit index (NFI), and incremental fit index (IFI) were equal to 0.839, 0.829, and 0.824, respectively. The minimum factor loading was 0.33 (item 8, Fever). The factor loadings of the other items were greater than 0.4. The structure diagram is shown in Figure 2.

**Figure 3:**
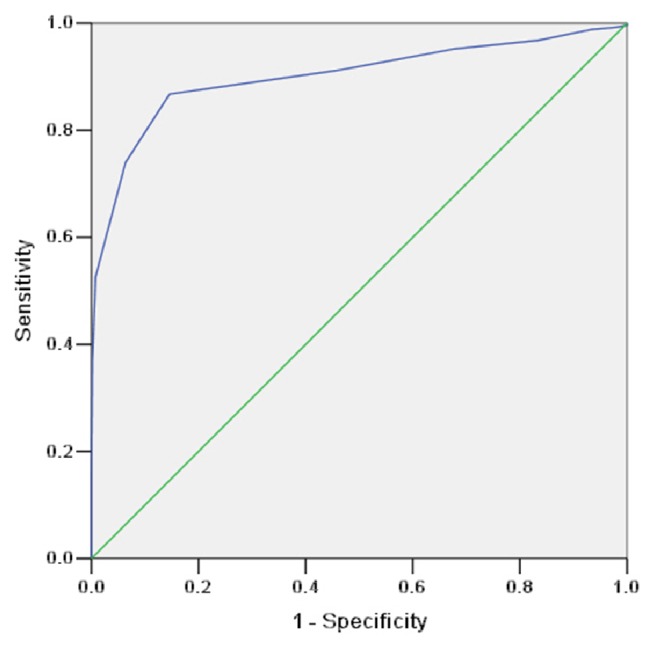
The ROC curve of large intestine dampness-heat syndrome.

The receiver operating characteristic (ROC) curve of the LIDHSQ was 0.900 (standard error = 0.014), with a 95% CI of 0.872–0.928 (*P* < 0.001). The ROC curve is shown in Figure 3. When the cut-off value of the LIDHSQ was ≥ 7, the sensitivity and specificity were 0.867 and 0.854, respectively (Table 7).

## 4. Discussion

The standard procedures raised by the WHOQOL group and the US FDA have been widely adopted to develop and validate TCM outcomes [22, 24, 25] and TCM diagnostic questionnaires [23, 26]. The WHOQOL group and the US FDA first proposed to establish the conceptual framework of the questionnaire. Chen et al. advocated using exploratory factor analysis (EFA) to explore the symptom domains of the kidney deficiency syndrome questionnaire [29]. EFA is merely a statistical analysis method in which the domains depend heavily on the included items and the factor extraction method. Differences in items or factor extraction methods will lead to inconsistent results. If relevant variables are omitted in the analysis, or scarcely reliable or redundant items are included, the final number and composition of the domains will be seriously affected [41]. However, the structures of the questionnaires based on the procedures of the WHOQOL group and the US FDA had high reliability and repeatability. Therefore, the procedures of the WHOQOL group and the US FDA were eventually utilized to develop the LIDHSQ.

The LIDHSQ had good content validity according to the review and suggestions of the experts. The LIDHSQ included the most important aspects that characterize LIDHS for the UC patients. The LIDHSQ contained 11 items. (1) The disease-related domain included diarrhoea, abdominal pain, bloody purulent stool, and mucus in the stool, which are the most common bowel symptoms in UC patients. Because UC patients with other syndromes may have had similar symptoms, these items were related to ulcerative colitis. (2) Among these items, fever, dry mouth, red tongue, yellow fur, and anal burning were used to assess heat syndrome. The first four symptoms (fever, dry mouth, red tongue, and yellow fur) are important items for diagnosing heat syndrome according to the theory of TCM. Anal burning could help to locate the intestine. (3) Greasy fur, diarrhoea, and defecation disorder were valuable for diagnosing dampness-heat syndrome in UC patients.

The LIDHSQ had good reliability and validity according to the results. The Kappa coefficients are categorized as very good (0.81 to 1.00), good (0.61 to 0.80), moderate (0.41 to 0.60), fair (0.21 to 0.40), and poor (< 0.20). When the Cronbach's alphas are between 0.70 and 0.95, the internal consistency is considered good [42]. When ICC is at least 0.70 in a sample size of at least 50 patients, the test-retest reliability is considered to be good [42]. All items had high Kappa coefficients (> 0.9) in our results, which indicated that the items had excellent consistency between the TCM doctors. The Cronbach's *α* of the LIDHSQ was 0.68, and those of all of the domains were greater than 0.6. The results showed that the LIDHSQ had acceptable internal consistency. The test-retest reliability coefficients were greater than 0.8. The RMSEA was equal to 0.090 (90% CI: 0.072–0.109), which showed that the LIDHSQ had good construct validity. The item fever (item 8) was common in UC patients with LIDHS. Although item 8 (fever) had low loadings (0.33), it was still kept in the LIDHSQ.

The LIDHSQ had good diagnostic efficacy. The cut-off value of the LIDHSQ was ≥ 7 according to the results of the ROC. Its sensitivity and specificity were 0.867 and 0.854, respectively. The LIDHSQ was sufficiently sensitive to discriminate the scores of UC patients with different syndromes. The scores of the LIDHSQ (including domains) were higher in the patients with LIDHS than those with other syndromes. These results were consistent with our hypothesis.

The aim of our study was to develop and validate the diagnostic criteria of the LIDHSQ, a questionnaire used in TCM practice. The questionnaire cannot be used to diagnose UC. Therefore, the patients enrolled in our study must first be diagnosed as having UC. It is feasible to establish a theoretical model of TCM syndromes based on the basic theory of TCM, literature review, and expert opinions. TCM syndromes can be treated as latent variables. Using a qualitative item bank, literature review, and expert consultation, we successfully developed the domains and items of the LIDHSQ. The structural equation modelling could be used to quantitatively test TCM syndromes, consistent with other studies [23, 29, 43]. Other researchers have also successfully used similar methods to study TCM syndromes. For example, suboptimal health status (SHS) was characterized by ambiguous health complaints, general weakness, and a lack of vitality. Yan YX et al. developed the SHS questionnaire [24, 44] and indicated that it was a reliable and valid questionnaire as a new instrument for predictive, preventive, and personalized medicine (PPPM) of TCM [45, 46]. The LIDHSQ, as a valid and reliable TCM syndrome scale, may also be applied to PPPM through patient diagnosis and therapy assessment.

There were some limitations in this study. (1) All of the patients in the study were enrolled from the First Affiliated Hospital and the Second Affiliated Hospital of Guangzhou University of Chinese Medicine. Most of the UC patients were from Guangdong Province. The LIDHSQ should be further evaluated using data from other geographical areas in China. (2) Only 249 patients with LIDHS were enrolled in this study. More patients with LIDHS should be enrolled to assess the validity, reliability, and diagnostic efficacy of the questionnaire. Only twenty-two inpatients were included for test-retest reliability. Terwee et al. suggested that ≥ 50 patients was adequate for the assessment of test-retest reliability [42]. Therefore, more patients with LIDHS should be enrolled to assess test-retest reliability. (3) The response options for the items were binary (positive and negative). The binary data could not describe the symptoms in detail. Multiple choice items should be considered for the future study of the LIDHSQ. (4) No objective (pathological) measures were provided for the diagnosis of LIDHS.

## 5. Conclusions

The LIDHSQ is simple and easy for TCM doctors to perform. The LIDHSQ is valid and reliable for measuring LIDHS in UC patients with good diagnostic efficacy. We recommend the use of the LIDHSQ in Chinese UC patients. Through establishing the LIDHSQ for UC and determining its cut-off value for diagnosis, we hope to diagnose TCM syndromes of UC in a standardized manner.

## Figures and Tables

**Table 1:** The 18 items from LIDHS diagnostic criterion for UC patients based on literature review.

Items	1998 criterion [17]	2002 criterion [18]	2005 criterion [19]	2010 criterion [20]	2011 criterion [21]
Diarrhoea	Yes	Yes	Yes	Yes	Yes
Abdominal pain	Yes	Yes	Yes	Yes	Yes
Bloody purulent stool	Yes	Yes	Yes	Yes	Yes
Mucus stool	Yes	No	No	Yes	Yes
Tenesmus	Yes	Yes	Yes	Yes	Yes
Anal burning	Yes	Yes	Yes	Yes	Yes
Fever	No	Yes	Yes	Yes	Yes
Scanty deep-yellow urine	Yes	Yes	Yes	Yes	Yes
Bitter taste in mouth	Yes	No	No	Yes	Yes
Dry mouth	No	No	No	Yes	Yes
Halitosis	No	No	No	Yes	No
Red tongue	Yes	Yes	Yes	Yes	Yes
Greasy fur	Yes	Yes	Yes	Yes	Yes
Yellow fur	Yes	Yes	Yes	Yes	Yes
Thick fur	No	No	No	No	Yes
Rapid pulse	Yes	Yes	Yes	Yes	Yes
Slippery pulse	Yes	Yes	Yes	Yes	Yes
Soft pulse	Yes	Yes	Yes	No	Yes

Yes: the item exists in the criterion. No: the item does not exist in the criterion.

**Table 2:** Characteristics of the included patients.

	Pilot study		Validation study	
	LIDHS (n = 80)	LIDHS (n = 249)	Other syndromes (n = 521)	*P* value
Age (years, $\overset{-}{x}\pm s$)	48.3 ± 16.9	46.4 ± 15.3	45.8 ± 16.2	0.655
Sex				
Male	50 (62.5)	152 (61.0)	239 (45.9)	< 0.001
Female	30 (37.5)	97 (39.0)	282 (54.1)	
Marital status				
Married	75 (93.8)	223 (89.6)	455 (87.3)	0.373
Unmarried	5 (6.3)	26 (10.4)	66 (12.7)	
Occupation				
Employed	28 (35.0)	92 (36.9)	156 (29.9)	0.254
Retiree	11 (13.8)	33 (13.3)	68 (13.1)	
Student	1 (1.3)	7 (2.8)	25 (4.8)	
Unemployed	26 (32.5)	70 (28.1)	155 (29.8)	
Unclear	14 (17.5)	47 (18.9)	117 (22.5)	
Smoking				
Yes	13 (16.3)	32 (12.9)	56 (10.7)	0.391
No	67 (83.8)	217 (87.1)	465 (89.3)	
Drinking				
Yes	7 (8.8)	24 (9.6)	45 (8.6)	0.649
No	73 (91.3)	225 (90.4)	476 (91.4)	
Dietary preference				
Yes	8 (10.0)	19 (7.6)	60 (11.5)	0.096
No	72 (90.0)	230 (92.4)	461 (88.5)	
Family history				
Yes	2 (2.5)	5 (2.0)	13 (2.5)	0.676
No	78 (97.5)	244 (98.0)	508 (97.5)	

LIDHS: large intestine dampness-heat syndrome.

**Table 3:** The results of the item selection (pilot study).

Items	p	*α* value	r	CFA	Reserved item
Diarrhoea	0.96	-0.06	0.49	0.35	Yes
Abdominal pain	0.96	-0.02	0.67	0.40	Yes
Bloody purulent stool	0.93	-0.12	0.62	0.71	Yes
Mucus stool	0.91	-0.19	0.69	0.70	Yes
Tenesmus	0.71	0.12	0.41	0.25	No
Anal burning	0.69	0.03	0.47	0.49	Yes
Fever	0.32	-0.08	0.56	0.56	Yes
Scanty deep-yellow urine	0.09	-0.02	0.32	0.09	No
Bitter taste in mouth	0.33	-0.16	0.67	0.41	No
Dry mouth	0.75	-0.16	0.65	0.51	Yes
Halitosis	0.04	-0.03	0.34	0.14	No
Red tongue	0.94	-0.03	0.45	0.73	Yes
Greasy fur	0.73	-0.04	0.55	0.25	Yes
Yellow fur	0.94	-0.01	0.46	0.70	Yes
Thick fur	0.32	0.05	0.47	0.26	No
Rapid pulse	0.60	0.11	0.24	0.29	No
Slippery pulse	0.51	-0.08	0.35	0.27	No
Soft pulse	0.36	0.07	0.23	-0.07	No
Defecation disorder	0.43	-0.05	0.60	0.34	Yes
Anorexia	0.33	-0.10	0.40	-0.24	No

p: the proportion of positive responses; *α* value: internal consistency coefficients; r: correlation analysis; CFA: confirmatory factor analysis. When the item meets two or more of the above criteria, it will be finally removed.

**Table 4:** The scores and correlation coefficients for each item (*n* = 249).

	Score of 0	Score of 1	Mean	Standard deviation	Kappa*∗*	Disease-related domain^∧^	Heat domain^∧^	Dampness domain^∧^
Diarrhoea	18	231	0.928	0.259	0.878	0.684	0.253	0.169
Abdominal pain	16	233	0.936	0.246	0.934	0.697	0.134	0.094
Mucus stool	28	221	0.888	0.317	0.926	0.751	0.153	0.090
Bloody purulent stool	29	220	0.884	0.321	0.904	0.749	0.117	0.121
Anal burning	73	176	0.707	0.456	0.940	0.185	0.650	0.030
Red tongue	25	224	0.900	0.301	0.923	0.224	0.561	0.118
Yellow fur	28	221	0.888	0.317	0.904	0.227	0.624	0.181
Fever	149	100	0.402	0.491	0.945	0.075	0.620	0.364
Dry mouth	61	188	0.755	0.431	0.928	0.053	0.704	0.053
Greasy fur	76	173	0.695	0.461	0.916	0.171	0.182	0.865
Defecation disorder	129	120	0.482	0.501	0.923	0.115	0.240	0.887

*∗*: Kappa was calculated using the scores assigned by the two experts. ^∧^: correlation coefficient between the item and domain scores.

**Table 5:** Cronbach's *α* coefficient and ICC of each domain.

	No. of items	Cronbach's *α* (95% CI)	ICC (95% CI)*∗*
Disease-related domain	4	0.69 (0.62–0.75)	0.86 (0.83–0.89)
Heat domain	5	0.61 (0.53–0.68)	0.83 (0.80–0.86)
Dampness domain	2	0.70 (0.61–0.76)	0.82 (0.78–0.86)
The LIDHSQ	11	0.68 (0.62–0.73)	0.88 (0.85–0.91)

ICC: intraclass correlation coefficient. *∗*: results based on 22 inpatients.

**Table 6:** Scores of UC patients with different syndromes.

	LIDHS (*n *= 249)	Other syndromes (*n *= 521)	*t* value (*P* value)
Disease-related domain	3.63 ± 0.83	3.15 ± 0.94	7.03 (< 0.001)
Heat domain	3.65 ± 1.27	1.32 ± 1.13	25.69 (< 0.001)
Dampness domain	1.18 ± 0.84	0.63 ± 0.58	10.43 (< 0.001)
The LIDHSQ	8.46 ± 2.06	5.10 ± 1.61	24.73 (< 0.001)

**Table 7:** Results of the diagnostic value test.

Gold standard	The LIDHSQ		All
	Positive (%)	Negative (%)	
Positive	216 (86.7)	33 (13.3)	249
Negative	76 (14.6)	445 (85.4)	521

Cut-off value of the LIDHSQ ≥ 7 was diagnosed as positive.

## Data Availability

The data used to support the findings of this study are available from the corresponding author upon request.

## Supplementary Materials

Supplementary Materials Table S1: the items of the large intestine damp-heat syndrome questionnaire.
