# Supplementary material for: Development of a Traditional Chinese Medicine Syndrome-Specific Scale for Ulcerative Colitis: The Large Intestine Dampness-Heat Syndrome Questionnaire
Source: Evid Based Complement Alternat Med. 2018 Jul 12;2018:4039019. doi: 10.1155/2018/4039019 (PMC6077564; doi:10.1155/2018/4039019)
Supplement: Supplementary Materials — Table S1: the items of the large intestine damp-heat syndrome questionnaire. [file 4039019.f1.doc]

**Supplemental material**

**Table S1. The items of the large intestine damp-heat syndrome questionnaire**

|  | No | Yes |
| --- | --- | --- |
| Item 1. Diarrhoea | 0 | 1 |
| Item 2. Abdominal pain | 0 | 1 |
| Item 3. Bloody purulent stool | 0 | 1 |
| Item 4. Mucus stool | 0 | 1 |
| Item 5. Fever | 0 | 1 |
| Item 6. Dry mouth | 0 | 1 |
| Item 7. Red tongue | 0 | 1 |
| Item 8. Yellow fur | 0 | 1 |
| Item 9. Anal burning | 0 | 1 |
| Item 10. Greasy fur | 0 | 1 |
| Item 11. Defecation disorde | 0 | 1 |

The scale was used by the doctors to diagnose the large intestine dampness-heat syndrome (LIDHS) of UC patients. Please fill in each item according to the conditions of the UC patients. The UC patients can be diagnosed as the LIDHS if their total score was ≥ 7. The detailed descriptions of each item are as follows:

Item 1 Diarrhea: refers to increased frequency of bowel movements, accompanied with thin, unformed or even watery fecal matter.

Item 2 Abdominal Pain: refers to pain in the abdomen.

Item 3 Mucous Stool: refers to feces containing mucus.

Item 4 Bloody Purulent Stool: refers to feces containing blood and pus.

Item 5 Anal Burning: refers to the burning sensation of the anus during defecation.

Item 6 Red Tongue: The tongue color is bright red, which is redder than normal one.

Item 7 Yellow Fur: the fur presents yellow color.

Item 8 Fever: refers to higher body temperature than normal one, or conscious sensation of systemic or local fever by patients themselves when body temperature is normal. The patients feel feverish without chills.

Item 9 Dry Mouth: refers to dry mouth.

Item 10 Greasy Fur: refers to small and compact granules on the surface of the tongue that are difficult to scrape off or exfoliate.

Item 11 Defecation Disorder: refers to unsmooth defecation. The patients feel obstructed and difficult to have a bowel movement.
